# Supplementary material for: Ulva compressa from Copper-Polluted Sites Exhibits Intracellular Copper Accumulation, Increased Expression of Metallothioneins and Copper-Containing Nanoparticles in Chloroplasts
Source: Int J Mol Sci. 2021 Sep 29;22(19):10531. doi: 10.3390/ijms221910531 (PMC8508654; doi:10.3390/ijms221910531)
Supplement: Supplementary file 1 [file ijms-22-10531-s001.zip › ijms-1385527-supplementary.pdf]

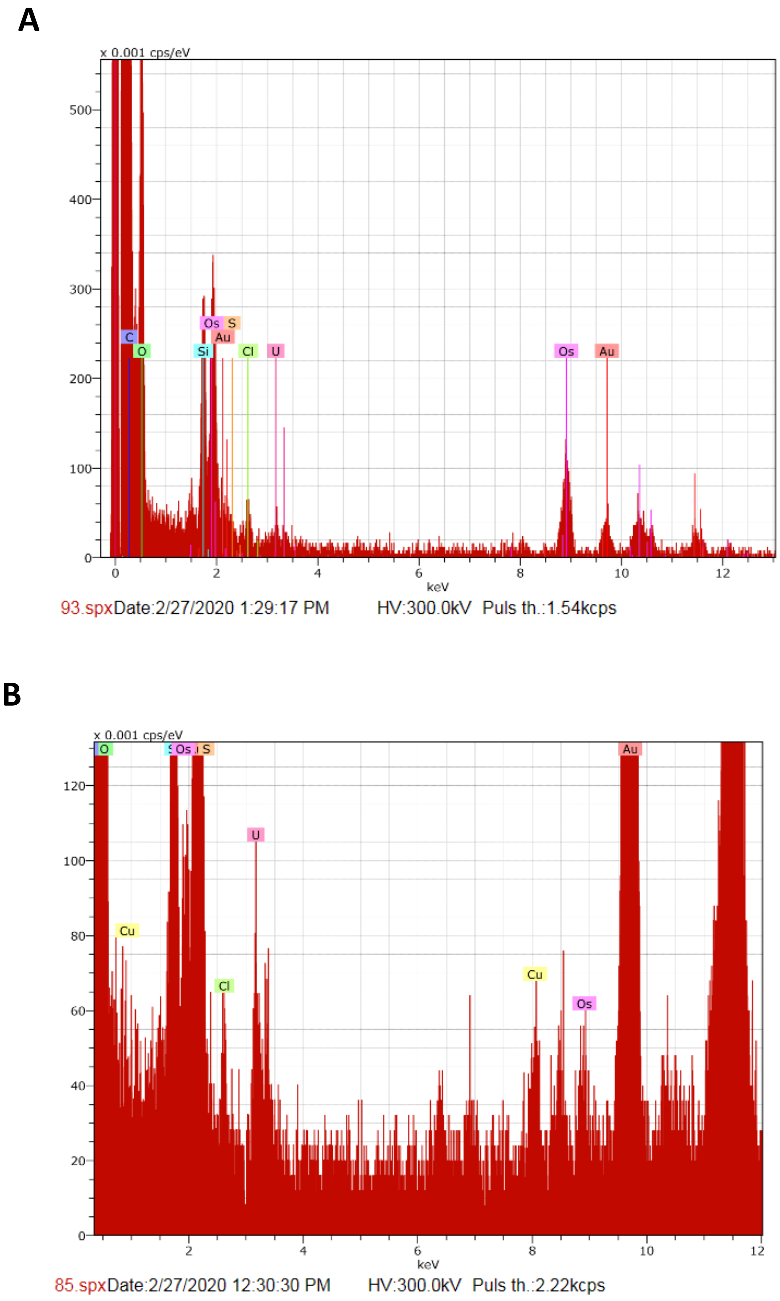

Figure S1. X-ray emission profiles of elementary metals detected by energy-dispersive x-ray spectrometry (EDXS) present in electrodense nanoparticles in a cell of *U. compressa* from a control site (**A**) and a copper-polluted site (**B**).
